# Account of a Method of Curing Burns and Scalds

**Published:** 1792

**Authors:** David Cleghorn

**Affiliations:** Brewer in Edinburgh.


XI. *Account of a Method of curing Burns and Scalds. By Mr.* David Cleghorn, *Brewer in Edinburgh. Communicated in three Letters to* John Hunter, *Esq. F. R. S. Surgeon Extraordinary to the King, Surgeon General to the Army,* &c., *and by him to Dr.* Simmons.

### LETTER I.

*To* JOHN HUNTER, *Esq.*

Edinburgh, 5th Auguſt, 1791.

SIR,

WHEN I had the pleaſure of ſeeing you in London in June laſt, I promiſed, upon my arrival here, to communicate to you my method of curing burns and ſcalds. I have been prevented by buſineſs from performing my promiſe ſo ſoon as I could have wiſhed, but I will now attempt it, and I hope I ſhall make myſelf intelligible to you.

I have already mentioned to you that my firſt application and moſt powerful remedy is vinegar. If the injury is on the fingers, hands, or lower parts of the arms, the application may very properly be made by an immerſion of the parts. Formerly I uſed alſo to immerſe the feet and lower ower parts of the legs, when injured, in a pail containing vinegar; but although no material bad consequence ensued from this practice, I found that, by placing the legs in a perpendicular posture, the sores were more apt to swell and inflame than when they were laid up and supported in an horizontal one. When, therefore, the feet or legs are injured, or when the injury falls on the thighs, the body, the face, or head, where immersion would be inconvenient or impracticable, the method I follow (and which I find very effectual) is to pour some vinegar into a plate or flat-edged dish, and to dip linen rags in the vinegar and lay them or let them drip on the sores. This operation of alternately dipping the rags and laying them on the parts affected, is repeated so quickly, that the parts are kept constantly wet, or rather overflowing with the vinegar, and the plates are so placed as to receive or catch as much of it as possible; and I continue to use what falls back again into the plates for some time, till it has become somewhat vapid, when I throw it out, and pour into the plates a new supply of fresh vinegar. I have known two English quarts of vinegar used in this way to a large scald on the legs in four or five hours; and if the sores have a large surface, and and are on the body, under which the plates cannot be ſo placed as to prevent it from ſpilling, a larger quantity ſtill of vinegar will be needed. So cooling and grateful are the effects of this application while any conſiderable degree of pain or heat remains, and ſo immediately does the uneaſineſs return when it is too early diſcontinued, that the patients themſelves ſeldom fail of giving their active aſſiſtance in this operation of wetting the parts affected.

In ſlight or ſuperficial injuries, by which I mean ſuch as are attended with no excoriation, but with pain, heat, or inflammation, and perhaps with ſmall bliſters, the vinegar, if early and conſtantly applied, is ſufficient to effect a cure without any other application. It almoſt inſtantly gives relief, and in two or three hours, and often in a much ſhorter time, the patient will be quite at eaſe. The application of the vinegar may then be intermitted; but as ſome degree of pain and heat will poſſibly return, and if not attended to might yet produce a ſore, the vinegar muſt be applied as often as any painful ſenſation returns; and to make ſure, it ought to be continued now and then for a day after. In ſhort, it is always prudent, even in theſe ſlight caſes, cases, to use the vinegar long and in abundant quantities.

In most instances, such slight injuries, as those I now speak of, are healed without ever breaking out into a sore; if, however, through neglect of using the vinegar speedily, or not continuing it long enough, and in sufficient quantities, from something peculiar in the patient's constitution, or any other cause, the injury should degenerate into a sore, it will readily be healed by the application of chalk and poultices in the manner hereafter to be mentioned.

In severe burns and scalds which have recently happened, and which are attended with large blisters, excoriations, or loss of substance, the vinegar must be constantly applied till the heat and pain nearly cease, which will happen in from two to eight hours, according as the injury is more or less severe. The sores must then be covered with rags or cloths well wetted, which, as often as they dry, or any sensation of pain or heat returns, must be wetted afresh with the vinegar for two, three, or four hours.

In the worst cases I have ever met with the pain became tolerable soon after the vinegar was applied, and in ten, or at most twelve hours, the patients patients were ſo much at eaſe, that in general they fell into a ſound ſleep.

When I firſt began this practice I uſed to keep the wetted rags on the ſores, without any other application, ſometimes for two or three days; but experience ſhewed me, that after the pain and heat peculiar to burns and ſcalds were removed, the vinegar excited ſmarting in the tender excoriated ſkin, and was in fact of no farther uſe: I therefore never employ it longer than twelve hours, excepting on the parts round the edges, or outſide of the ſores, which I foment with it for a minute or two before the dreſſings, to be afterwards mentioned, as long as they continue in any degree ſwelled or inflamed.

The wetted rags being removed, the ſores muſt next be healed with other applications; and the firſt dreſſing I uſe is a common poultice made of bread and milk, with a little ſweet oil or freſh butter in it. I lay the poultice cloſe to the ſore, and uſe no gauze or cambric between them. The firſt dreſſing ſhould remain ſix, or, at moſt, eight hours, and when it is removed the ſores muſt be covered entirely with chalk finely pounded or ſcraped (for, inſtead of pounding the chalk, I generally hold a lump of it over the ſores, and ſcrape it with a knife upon them) till the powder has abforbed the matter or ichor from the fores, and appears quite dry all over them. A frefh poultice is then laid over the whole, and the fame fort of dreffing with chalk and poultice is repeated morning and evening till the fores are healed.

In fome cafes, after the fecond or third day, if the fores are on a part of the body where it is difficult to keep the poultice from fhifting, I ufe, inftead of it, a plafter, pretty thickly fpread, of the common white lead ointment through the day, (covering the fores previoufly with chalk) and chalk and poultices through the night as already directed. I alfo ufe the fame kind of white ointment, occafionally, through the day, when I think the conftant renewal of poultices has foftened and relaxed the fores too much; a circumftance which, notwithftanding the abforbent quality of the chalk, will, at times, in fome degree happen.

In cafes where there are large blifters, before I apply the vinegar, I open them with a pin or a lancet in different parts, and gently prefs the water out of them with a linen cloth. The intention of this is to bring the vinegar to act more clofely upon the burnt flefh, and I have found it to have an excellent effect.

Whilft

Whilſt any of the ſkin of the bliſters remains on the ſore, matter will form and lurk under it, which cannot be reached and abſorbed by the chalk. New punctures, therefore, muſt be made at every dreſſing, whenever matter (which muſt be gently preſſed out with a cloth) is ſeen lurking, and as ſoon as the ſkin has loſt its toughneſs ſo much that it can be ſeparated from the ſore without irritating it, which in general is the caſe on the ſecond or third day, it ought to be gently and gradually picked off when the ſores are dreſſed, and plenty of chalk inſtantly laid on to prevent any bad effects the air might have on ſores in a ſtate ſo highly ſuſceptible of injury.

In ſevere caſes, or ſuch as are attended with excoriation or loſs of ſubſtance, when the vinegar is not applied within twenty-four hours of the time the accidents happen, it almoſt always gives conſiderable pain; but if the patient can endure it, the ſores may ſafely be wetted all over for a quarter or half an hour, or even much longer. The ſmarting is no doubt a little irkſome, but it is worſt at firſt, and, at any rate, goes off immediately upon diſcontinuing the vinegar, and leaves the ſores in a much cooler or leſs inflamed ſtate. If the patient, however, cannot or will not bear the vinegar on the raw and tender parts of of the ſore, I then cover thoſe parts cloſe with a plaſter of the white ointment, and wet all round them with the vinegar for a quarter or half an hour, or longer. The ointment is then taken off, and the ſores are covered with the powdered chalk, and a poultice laid over all; and they are afterwards to be treated, in all reſpects, till they heal, as the ſeverer ſort of ſores, to which the vinegar has been early applied, are already directed to be, after the pain and heat have left them.

The vinegar I prefer is that made of the beſt white wine; but any ſort that has enough of acid will anſwer, provided there be no admixture of any mineral acid. In ſevere caſes I generally warm the vinegar, before I uſe it, to nearly blood heat, eſpecially in cold weather, and where a great deal of it muſt be employed. When it is applied cold, and in great quantities, it is apt to bring on a chilneſs and ſhivering, which I have always removed readily, by wetting the feet with cloths dipped in warm water, and giving the patient a little warm water to drink, with ſome ſpirits added to it ſo as to be rather ſtronger than good punch. If the arms or hands are badly injured, I keep them, during the cure, always ſlung; and if the legs, I endeavour to ſupport them ſo as as to procure as much eafe to the patient as poffible.

I am, with much refpect, &c.

---

### LETTER II.

Edinburgh, 3d October, 1792.

SIR,

AS I did not, in my letter of the 5th of Auguft, give you fo full an account of my method of curing burns and fcalds as I intended, I fhall now trouble you with fome additional obfervations on the fame fubject.

When I was fpeaking of the feverer fort of injuries, I omitted to mention that they are more eafily cured on the face than on any other part. In the worft I have met with I have never had occafion to ufe the chalk and poultices, and yet complete cures were always effected without leaving mark or fcar; and this is fo far fortunate, as the application of poultices to fome parts of the face would be very inconvenient. Were the face, however, to be fo deeply burnt as to occafion a confiderable lofs of fubftance, or were the injury, though trifling at firft, (through neglect of the ufe of vinegar) to break out into an ulcer or bad had fore, (a cafe which I never happened to meet with) chalk and poultices, or chalk and white ointment, would no doubt be neceffary.

The cafes that have occurred to me of injuries of this fort on the face, were not of the worft kind; but fome of them, notwithftanding, were pretty fevere; and as it is of particular importance to prevent fcars on that part, I fhall felect three of them, occafioned by different forts of accidents, and ftate how I treated them.

The firft I fhall take is that of a boy, of four year old, who fell with his face upon a room grate pretty much heated, and left on one of the bars of it the fkin of both his lips, and of one of his cheeks to the ear. The vinegar was inftantly applied (cold), and continued without interruption for about four hours, during which time the boy complained very little of pain. The accident happened at fix o'clock in the evening, and by nine he was quite eafy; about ten he fell afleep, and did not awake till morning, although during the night the vinegar was twice applied. In the morning he was free of pain, and continued fo. The parts, however, were frequently wetted with vinegar for three days longer, and afterwards anointed with white ointment four or five times a day. A thin blackifh-coloured in-

 cruftation cruſtation formed, (without the leaſt appearance of matter) which gradually peeled off, and in nine days after the accident happened, a new ſkin, perfectly ſmooth, was ſeen over all, which could only be known from that on the reſt of the face by its being of a deeper red.

The ſecond caſe I ſhall mention is that of a young man whoſe face was burnt by the exploſion of gunpowder. The whole ſkin under his chin and about the ears, of the eyelids, lips, cheeks, &c. was either in bliſters or peeled off. Cold vinegar was immediately applied to the eyelids, (which the patient kept ſhut) as well as to every other part of the face, and it gave immediate relief. The accident happened about eight o'clock at night, and the vinegar was continued till twelve, when the patient was ſo eaſy that he fell aſleep. He awoke once or twice in the night, and wetted his face a little with the vinegar himſelf, and in the morning he was quite free of pain, and continued ſo.

The vinegar was applied for two days longer, and the parts were alſo for thoſe two days, and ſome days after, anointed three or four times a day with a liniment made of linſeed oil and lime water, and by turns, as often with the white ointment. No part of the ſores ſhewed any tendency to fefter. New fkin formed on different parts without any incruftation, and on other parts a thin blackifh one (as ufual) formed, which, in fix days from the accident, fcaled off, and new fkin appeared over the whole face, and not the fmalleft mark was to be feen, excepting the rednefs of the fkin peculiar to all newly-healed fores.

The third and laft cafe is that of a boy of five years old, who was leaning on a fmall cord which hung acrofs the front of the chimney for drying clothes, and the cord giving way, the boy fell forwards, and his whole face plunged, from ear to ear, into a pot of water actually boiling on the fire. The accident happened on a Sunday, at one o'clock of the afternoon, and till I faw him, which was on the Tuefday following, about noon, the parts had been anointed with linfeed oil. By this time his face was fo much fwelled, (particularly his eyelids) and fo disfigured with fcabs and blifters, that the features of a human creature could fcarcely be diftinguifhed. The eyes were entirely clofed and, as I concluded the boiling water muft have got into them, I never expected he would fee again.

His ſkin was hot, his pulſe feveriſh; he had eaten very little; had got no ſleep ſince the accident; was exceedingly fretful, and in great pain.

He was brought to my houſe from a place at ſome diſtance, and was cold and chilly. I placed him near a good fire, ſo as to warm him, particularly his feet. At the ſame time the vinegar was applied (cold) to his face, without intermiſſion, for three quarters of an hour. By this time the patient was conſiderably relieved, and he was carried home, and laid in bed, where the vinegar was conſtantly applied for two hours more, when he fell aſleep, and did not awake for three hours, although the parts (excepting the eyes) were ſeveral times wetted while he ſlept. The vinegar was afterwards continued, in the uſual way, till ten o'clock, and the patient being coſtive, I ordered him ſome boiled pot barley and prunes, which he ate heartily. He ſlept pretty ſoundly all night, in the courſe of which his face was twice or thrice wetted. Next morning he was cool, the ſwelling of his face was greatly ſubſided, and the pain had abated ſo much, that he was no longer fretful or peeviſh. The vinegar was uſed pretty frequently this day (Wedneſday), and the ſores were, beſides, three or four times anointed anointed with the liniment already ſpoken of, care being taken not to lay it ſo thick on the eye-lids as to make it run into the eye, leſt the lime in it ſhould injure his ſight. He reſted well this night, and on Thurſday morning he was quite eaſy, and I was happy to find that he could ſee a glimmering of light with both eyes.

The vinegar and liniment were alternately uſed, now and then, during the whole of Friday, when the ſwelling of the face was almoſt quite reduced, and the patient could ſee perfectly with both eyes.

On Saturday and Sunday the liniment and white ointment were uſed, by turns, ſeveral times, and on Monday the cure was complete, nothing then remaining on the face but a little incruſtation, which went entirely off in a few days, and not a mark remained, nor the leaſt weakneſs or ſoreneſs in the eyes.

From the liniment having been uſed in two of the above caſes on the face, inſtead of the chalk and poultices, and the cures having been ſo immediate and complete, it might be thought, perhaps, that it would be as effectual as they are, were it uſed in their ſtead on bad ſores on any part of the body. To aſcertain this I made a number of experiments, and found that it had not the effects of the chalk and poultices. When fores were raw and relaxed, on any part of the body or extremities, anointing them with the liniment juft previoufly to the dreffing with the chalk, &c., was of fervice in bracing the parts, and I therefore ufe it now on fuch occafions. But in one inftance of a bad fcald on the feet, after ufing the vinegar in the ordinary way till the pain had ceafed, I ufed the liniment by itfelf; and although the fore had the appearance, in a fhort time, of clofing aad healing, there remained matter lurking within, which afterwards broke out, and the fores became worfe than ever. I was therefore obliged to ufe poultices without chalk, for two dreffings, to dilate the fore, and then to heal it with chalk and poultice in the ufual way.

The liniment does not appear to be abfolutely effential even on the face; for befides the firft of the three cafes juft mentioned, where it was not ufed, and the cure was complete, I have had many other inftances of the fame kind; and upon one occafion, when two young men were badly burnt all over their faces by the fame explofion of gunpowder, I firft applied, to the one, vinegar till fuch time as the heat and pain had quite abated, abated, and afterwards the liniment by itſelf; and to the other I uſed the vinegar for two days, and afterwards the white ointment, and they were both healed at the ſame time. As the liniment, however, is neither expenſive nor troubleſome, either in the mode of preparing or applying it, and as I am rather inclined to think that it is of ſome ſervice on the face as well as on a raw ſore, I, of late, always apply it to both in the way I have already mentioned.

It was owing to my being told by profeſſional gentlemen (to whom I was recommending my application) that this liniment was more efficacious in curing burns than any of my remedies could be, that I was induced to make many comparative trials of it with my own method. One caſe, in which I had by accident an opportunity of proving the inferiority of the liniment, being curious, and as I deviated in it a little from my ordinary mode of practice, it may not be amiſs to lay it before you.

A brewer's ſervant was ſcalded on his back and both legs with hot wort, (not boiling.) I was aſſured there were at firſt only a few ſmall bliſters on his legs, and that his back was only red and inflamed. I am confident, therefore, that if the vinegar had been applied immediately, the cure would have been effected in a ſhort time, and perhaps without any ſore. The liniment, however, was applied, and continued for eight or nine days. The patient, as I afterwards learned from himſelf, found no relief from it; every part of his legs and back, which the wort had reached, broke out into ſores, and at the end of nine days after the accident, when I, at the deſire of a friend, went for the firſt time to ſee him, I found him in great diſtreſs. The ſkin was entirely off his legs (excepting behind where the wort had not reached) from the knees to the toes, and in ſome parts the ſores were badly ulcerated, and diſcharged a thin ichor. The under part of the back, and from thence nearly to the anus, was excoriated. It was not, however, ulcerated like the legs, for the patient's waiſtcoat having been on when he met with the accident, had ſaved his back in ſome degree.

His pulſe was quick, but feeble; he had taken ſome purgative medicine, which obliged him to riſe frequently to go to ſtool, and he was ſo languid, that I thought it neceſſary to give him ſome cordial before I began my operations. As I underſtood that he had drank pretty freely when in health, I gave him nearly a glaſs full of brandy, brandy, diluted with warm water, which recruited his ſpirits greatly: I then covered the whole ſores on the legs thick over with ſcraped chalk till the ichor was entirely abſorbed, and laid poultices over all. This was no ſooner done than the patient ſaid he felt himſelf eaſier.

Here I would not make the vinegar my firſt application, becauſe the ſores on the legs were ſo exceſſively irritable, that the patient could not have endured the ſmarting it would have occaſioned; neither could I attempt covering them with plaſters of the white ointment till I had bathed the edges or outer parts of the ſores, (as I have ſaid I do on ſuch occaſions) for the moſt gentle touch would make them bleed, and the plaſters could not have been laid on and taken off without conſiderable irritation. I therefore deviated from my ordinary practice, by uſing the chalk firſt, as already related; and the event proved that, unaſſiſted by the vinegar, it has powerful effects, which I have on other occaſions alſo experienced.

The ſores on this patient's back, as I have already obſerved, were not ſo bad as thoſe on his legs; they were, however, raw, much inflamed, and painful; and as, owing to the ſtate and ſituation of the ſores on his legs, he could not not lye in any other pofture than on his back, his fores there gave me at this time moft anxiety. I applied the vinegar to them (warmifh), which occafioned at firft a pretty fevere fmarting, but after being continued for fome time, the parts became lefs fenfible, and the patient felt very little pain. The vinegar was ufed for about a quarter of an hour; the parts were then covered with chalk, and a plafter fpread thick with white ointment was laid over all. I fhould have preferred poultices to white ointment, had I not thought they would have been more apt to be fqueezed and fhifted by the weight of the body.

As the feafon was cold, (January 13th) I wrapped flannel cloths round the patient's legs and feet, above the dreffings, and left him about one o'clock greatly relieved. I vifited him again at nine at night, and found him pretty eafy. He had got fome fleep; the fores on his legs had a more kindly appearance; and as they were not fo very tender and irritable as before, I ventured to wet all round them with vinegar, and then covered them again with chalk and renewed the poultices.

The fores on the back, which were remarkably better, I bathed well with vinegar for five or fix minutes, and then dreffed them with chalk chalk and ointment as before. I continued the ſame treatment to the back, morning and evening, and on the 16th, at night, the patient could lye on it without pain. A thin incruſtation formed on the parts and they were completely whole on the 23d.

I had directed that the legs ſhould be dreſſed every morning and evening with the poultices and abundance of chalk, whether I was preſent or not; but the patient's wife entertaining a prejudice againſt the chalk, (in conſequence, as ſhe afterwards told me, of a converſation ſhe had had with a ſtudent of phyſic) ſhe omitted uſing it every time I was abſent at the dreſſings, which happened to be the caſe the three mornings juſt preceding the 28th, and on that morning I obſerved the ſores (though greatly contracted) were too moiſt and relaxed; there was on ſeveral parts of them ſome blackiſh clotted blood, probably owing to the legs rubbing on one another in ſleep; the parts too were a good deal inflamed, and the patient complained of pain. On this occaſion, with a view to invigorate and brace the parts, I wetted the ſores twice or thrice with brandy, and then dreſſed them with chalk and white ointment in the uſual way. The patient felt himſelf eaſy and comfortable, and from this time the cure went rapidly on, the chalk having no more been omitted. The dreſſings with it and the white ointment were continued, morning and evening, and beſides, on two days, viz. the 28th and 29th, about two o'clock of the afternoon, the ſores were covered over with chalk, without renewing the ointment.

On the 30th all was in a manner whole, excepting two parts, rather leſs in ſize than a ſixpence, upon which ſome proud fleſh had grown, and threatened to leave a ſcar. Theſe parts were, previouſly to three or four dreſſings, powdered over with burnt alum, and dry ſcabs formed on them, which gradually diminiſhed, but were not entirely off for above two weeks. The cure, however, may be ſaid to have been completed on the 7th of February, when the man went to his work, exactly twenty five days from the time I firſt ſaw him. Diachylon plaſter was uſed to the ſcabs after the man went abroad to work, till they fell off, and no ſcar, nor mark, except the uſual redneſs, remained.

Dr. James Hay, Fellow of the College of Phyſicians of Edinburgh, (a gentleman of extenſive obſervation and experience, and of too much candour of mind and liberality of ſentiment timent to think that a valuable diſcovery in the healing art ſhould be diſregarded, and the benefit of it loſt to mankind, merely becauſe it happens to be ſtumbled on by a perſon belonging to none of the medical profeſſions) has always paid particular attention to the accounts I have given him of my cures, and has condeſcended, upon ſeveral occaſions, to viſit my patients, and to ſee with his own eyes the effects of my applications. In particular, he viſited the laſt-mentioned patient again and again, and took the trouble of reconciling his wife to the chalk, which ſhe had been told, as it checked the ſuppuration, would be attended with fatal conſequences. The doctor, was chiefly ſurpriſed in this caſe at the rapid cure of the back, notwithſtanding the patient was obliged to lye on it, and he gratified my feelings not a little by ſaying, that, in the common way of treating ſuch a ſore, he doubted much if a mortification of the back could have been prevented, and that it was his opinion I had ſaved the man's life.

If my remedies really were the means of ſaving this patient's life, in the ſeventeen years practice I have had, I muſt have been acceſſary in ſaving the lives of others, for ſeveral of my patients were in a much worſe ſtate than this.

Beſides

Befides many people fcalded with boiling water, broth, ftarch, &c., I have cured a variety of burns occafioned by melted lead and brafs, liquid pig iron, red hot bar iron, the flames of fpirits, burning coals, linen, &c. quick lime, and by the explofion of gunpowder: and there is no part of the body that one or other of my patients has not been burnt or fcalded on.

One child, in going backwards, was thrown down by a pot ftanding on the floor, newly taken off the fire, and almoft full of boiling broth, and fell into, or rather fat down in it, and fcalded, in a very bad manner, his anus, fcrotum, and parts adjacent, but was healed in a furprifingly fhort time, the vinegar having been early applied: and a Blackfmith once was relieved and cured, who was in great agony from a fpark of hot iron which flew into his eye from a piece he was ftriking on an anvil. In this cafe the vinegar was diluted with water to one half of its ftrength, and the patient let fome of it into the eye. He alfo kept the eye fhut, and bathed it with vinegar of a full ftrength.

In what manner my applications act, fo as to prevent marks and fcars, I do not pretend to explain; but I uniformly obferve that, when ufed in time, they entirely check fuppuration in all flight cafes, and that even in many fevere ones pus or matter is hardly ever feen. In deep burns too, attended with lofs of fubftance, the difcharge muft appear aftonifhingly little to thofe who have been accuftomed to fee fores cured in the ordinary way. It has been commonly remarked, that burns and fcalds fpread or enlarge for eight or ten days; but with my treatment they vifibly contract from the beginning. The new fkin begins to form round the extremities of even a bad fore fometimes fo early as the fecond day, and in the middle, where there has been a lofs of fubftance, the new flefh fhoots up from the bottom rather with a fungous appearance, the furface of it being unequal, fomewhat refembling heads of pins or the candying of honey, (but of a flefh colour) and continues gradually to grow till it rifes to the height of the found flefh around it, when the fkin forms at once without incruftation. When I began my practice indeed (I do not fpeak here of the face, my treatment of it and the effects thereof having been always much the fame) I ufed the vinegar in bad cafes much longer than I do now, and did not apply the poultices for twenty-four hours, or or oftentimes more; a dry ſcab, ſtained by the vinegar of a black ink colour, (eaſily accounted for) would then form over all the excoriated places, and under it there was always matter. The poultices which were then applied brought off the ſcab generally in a lump the third or fourth dreſſing, and a very tender bleeding ſore was thus expoſed, which I inſtantly laid very thick over with ſcraped chalk and poultices. After this, the very ſame method was obſerved which I now follow, and the ſores healed without a ſecond ſcab or incruſtation, and without mark or ſcar, as they do now. As I know little of theories, I cannot ſay whether theſe circumſtances, when duly conſidered, will confirm, or contradict, or throw any new light on the received opinion concerning the uſe of ſuppuration in the production of new fleſh; but this I can ſafely affirm, that I have neither advanced any thing that has not actually happened in the courſe of my long experience, nor have I exaggerated, to my knowledge, any of the circumſtances of the caſes I have related, as I truſt you will in due time be convinced of from your own experience.

With regard to diet, I allow my patients to eat boiled or roaſted fowl, or, in ſhort, any plain dreſſed dreffed meat they like; and I do not object to their taking (with moderation, however) wine, water and fpirits, ale, or porter. My applications, as hath been already obferved, allay pain and inflammation, and alfo always either prevent or remove feverifhnefs; and as at the fame time (if one may judge from their effects) they have powerful antifeptic virtues, I have never had occafion to order bark, or any internal medicines whatever, and I have only once thought it neceffary to let blood. When a patient is coftive, I order boiled pot barley and prunes, or fome other laxative nourifhing food, and fometimes an injection, but never any purgatives. It is diftreffing to a patient with bad fores to be often going to ftool. Befides, I have remarked that weaknefs and languor (which never, in my opinion, haften the cure of any fore) are always brought on more or lefs by purgatives. From the effects too I have felt them have on myfelf, and obferved them to have on others, they do not feem to me to have fo much tendency to remove heat and feverifhnefs as is generally imagined; and I fufpect that, contrary to the intention of adminiftering them, they oftener carry off ufeful humours than hurtful ones. But I am going out of my depth, and expofing myfelf to criticifm, by fpeaking on a fubject that I furely muft be very ignorant of; I will, therefore, return to my vinegar. I have already faid that I always prefer wine vinegar, when it is to be had; I have, however, ufed, with very good effect, vinegar made of fugar, goofeberries, and even alegar; but which ever of them is taken, it ought to be frefh and lively tafted.

I once made fome trials (on a burn I met with myfelf) of oil of vitriol diluted with water and of different degrees of ftrength, but I found its effects to be the very reverfe of vinegar, for it increafed the pain and heat even when it was pretty much diluted. I make no doubt that diftilled vinegar might do; but fince the common fort, when frefh and good, has in every cafe been fo efficacious, there feems to be no occafion to attempt improving upon it; and as acids are of a pungent penetrating nature, perhaps it would not be fafe to apply one too ftrong to a raw and tender fore. Even the common vinegar, only by being ufed too cold, affected two of my patients with tremblings and chillinefs, which alarmed me a good deal. I removed thefe fymptoms indeed (as I

before before mentioned), very readily, by warming the patients feet with cloths dipped in warm water, and giving them warm water and ſpirits to drink; but ever ſince I have been careful to uſe precautions againſt the like ſymptoms, particularly in cold weather, by warming the vinegar a little, placing the patients near a fire, giving them ſomething warm internally, and, in ſhort, by keeping them in every reſpect in a comfortable condition.

In any ſlight caſe it is not neceſſary to heat the vinegar, and ſeldom in ſevere ones, if the injury is on the hands or face. Were it not from its chilling effects, it ought to be uſed cold on every part, becauſe heating weakens it, and haſtens its becoming vapid during the application; when uſed warm, it muſt, therefore, be the oftener thrown out and replaced with a freſh ſupply.

If the vinegar is introduced into hoſpitals, tubs (reſembling bathing tubs, but ſhallower) that would hold a patient at full length would be uſeful in caſes of univerſal burns or ſcalds. A mattreſs, or ſomething ſoft, ſhould be made to fit the tub, and the patient ought to be extended on it, and as much warm vinegar poured into the tub as would wet all the under part of the body and the ſides, and the upper part might be wetted with cloths. I never met with ſuch a caſe; but from the ſucceſs I have uninterruptedly had, I ſhould not be afraid of undertaking almoſt any caſe.

Before I conclude I muſt obſerve, that, upon looking over my caſes, I ſee I generally do not open the bliſters till the vinegar has been applied for an hour. I ſaid in my laſt that I opened them before I began to uſe the vinegar, but I was then in a miſtake; it is, however, I believe, not very material.

I have now, Sir, ſaid every thing on this ſubject that occurs to me to be neceſſary. I wiſh I could have been leſs prolix; all I have aimed at is to be underſtood, and I wiſh I may have ſucceeded even in that: I ſhall, however, at all times be proud to anſwer you punctually any queſtions you may put to me on points on which I have not been intelligible, or on any new ones that may occur to yourſelf; and I flatter myſelf that, under your foſtering hand, this accidental diſcovery of mine may one day be conſidered as no unimportant acquiſition to the healing art.—I converſed with you, when in London, about the poſſibility of extirpating old ſcars, and I intend, when opportunities offer, offer, to make ſome experiments on this ſubject, the reſult of which I ſhall take the liberty of communicating to you.

I have the the honour to be, &c.

---

### LETTER III.

Edinburgh, 4th November, 1791.

SIR,

IT is my earneſt wiſh that the purport of what I have written to you on the ſubject of burns and ſcalds ſhould be made public, and nothing, therefore, could give me more pleaſure than to ſee, by your letter of the 19th of October, that you ſeem to think my communications are not unworthy of a place in the Medical Facts and Obſervations.

What I mentioned in my laſt letter concerning the poſſibility of eradicating old ſcars, is, at preſent, merely matter of ſpeculation, and if taken notice of at all in the intended publication, ſhould, I think, only be mentioned by way of hint. The method I propoſe to myſelf for filling up and effacing old ſcars, and removing claret and other marks children are born with, is to bliſter the parts, and then lay iſſue plaſters on them till there is a complete excoriation and a ſuppuration begun, when I would heal the ſores with chalk and poultices, as in burns.

I do not expect that in every caſe this will be effectual, but I ſhould imagine it may be tried with ſafety, and that probably it may in many caſes be of ſervice.

As I wiſh not to advance a ſingle circumſtance that is not ſtrictly fact, I have, ſince I received your letter, viſited a good many of my old patients with a view of examining again whether any marks or ſcars really remain on the parts that were affected, and I think it proper to inform you that not a veſtige of a mark is to be ſeen on any of their faces, and in general on no other part, though I muſt confeſs I found ſome exceptions. One boy had a pretty large ſcar on his foot, deeper than the pits of the ſmall pox, but without any ſeams or ridges on it, neither is it in the leaſt drawn together. This boy was very unruly, and never could be kept from running and jumping about during the cure, which, although the injury was not a ſevere one, was the moſt tedious of any I have ever met with. There was probably ſomething unfavourable vourable in his conftitution, as he once before had a fore on the fame foot that was very obftinate, and left a deep hole.

The fkin on the parts affected of the brewer's fervant, whofe cafe I related in my laft, feems thinner than that on the other parts of his legs, and has not regained its proper colour, but the furface is fmooth and even with the flefh around. A man too who was burnt with liquid pig iron on both his legs and feet, in a moft fhocking manner, and whofe cafe was the worft I have ever encountered, bears fuch marks as readily fhow where the worft of his fores were: there are, however, no pits or hollow places, and but little rednefs on the fkin, which appears fomewhat clear and thin, and the lines on the furface do not run in the fame direction with thofe on the fkin around, but are drawn as if from the center to the extremities of the parts that were affected. The two laft-mentioned patients are hard-working men, in poor circumftances, and could not afford to give their legs eafe fo long as I could have wifhed: they took little care too to keep foft linen between their hard coarfe worfted ftockings and the tender fkin when newly formed, and were not in many other refpects treated and taken care of, when the fores were at the worſt, as patients would have been in better circumſtances; all which may perhaps account, in ſome meaſure, for the appearances I have deſcribed.

Before I conclude I ſhall take the liberty to ſuggeſt, that my method of cure ſhould be particularly recommended to the ſurgeons of the army and navy, where accidents by the exploſion of gunpowder have often been fatal. The flame of it paſſes inſtantly off the parts, and conſequently does not burn them deep; and I have, on that account, always found leſs difficulty in curing injuries by gunpowder than almoſt any others.

I am, with much reſpect,

SIR,

Your moſt obedient, humble ſervant,

DAVID CLEGHORN.

